# Significance of serum palmitoleic acid levels in inflammatory bowel disease

**DOI:** 10.1038/s41598-021-95923-6

**Published:** 2021-08-10

**Authors:** Yuko Akazawa, Tomohito Morisaki, Hiroko Fukuda, Kiyuu Norimatsu, Junya Shiota, Keiichi Hashiguchi, Maiko Tabuchi, Moto Kitayama, Kayoko Matsushima, Naoyuki Yamaguchi, Hisayoshi Kondo, Fumihiko Fujita, Hiroaki Takeshita, Kazuhiko Nakao, Fuminao Takeshima

**Affiliations:** 1 Tissue and Histopathology Section, Atomic Bomb Disease Institute, Nagasaki University, Nagasaki, Japan; 2 JCHO Isahaya General Hospital, Isahaya, Japan; 3 Sasebo City General Hospital, Sasebo, Japan; 4 Department of Gastroenterology and Hepatology, Nagasaki University Graduate School of Biomedical Sciences, Nagasaki, Japan; 5 Biostatistics Section, Division of Scientific Data Registry, Atomic Bomb Disease Institute, Nagasaki University Graduate School of Medicine, Nagasaki, Japan; 6 Department of Surgery, Kurume University, Kurume, Japan; 7 NHO Nagasaki Medical Center, Nagasaki, Japan; 8 Nagasaki Goto Chuoh Hospital, Goto, Japan

**Keywords:** Diseases, Gastroenterology

## Abstract

Inflammatory bowel diseases (IBDs), including ulcerative colitis (UC) and Crohn’s disease (CD), are chronic intestinal diseases of unknown etiology that present with variable disease extents and outcomes. The use of biomarkers for the diagnosis and management of IBDs is considered beneficial. Palmitoleic acid (PO) is an adipose tissue-derived mono-unsaturated free fatty acid that potentially serves as a lipokine in metabolic and inflammatory diseases. The aim of this study was to investigate the significance of PO levels in the serum of patients with UC and CD. The study included patients with UC (n = 22), patients with CD (n = 35), and controls (n = 22). The levels of serum PO were analyzed using gas chromatography. The association of serum PO levels with the clinical features and disease outcomes in IBD was examined. Serum PO levels were significantly higher in patients with CD than in controls, whereas no difference in these levels was observed between patients with UC and controls. Serum PO levels were significantly associated with the CD activity index. Additionally, high serum PO levels were associated with an increased risk of surgical intervention requirement during follow-up. In a pilot study with a few patients, high PO levels were observed in the mesenteric tissue in the active disease site of patients with CD (n = 7) compared with those with colon cancer (n = 6). Elevated serum PO levels might serve as a marker for local inflammation and prognosis in patients with CD.

## Introduction

Ulcerative colitis (UC) and Crohn’s disease (CD) are inflammatory bowel diseases (IBDs). They exhibit similar clinical manifestations; however, only the mucosal layer in the large intestine is affected in UC^1^, whereas the entire gastrointestinal system may be involved in CD^1^. Both diseases often require long-term treatments, including 5-aminosalicylic acid (5-ASA), immune-suppressor, and anti-tumor necrosis factor-alpha (TNF-α) administration. Failure to control flare ups and induce remission with drug therapy can necessitate surgical interventions, including colectomy, enterectomy, and abscess drainage^2^. The severity and persistence of IBD markedly vary among the patients; it is difficult to assess the prognosis of the disease, especially, the risk for surgical intervention. Therefore, identifying a potential biomarker for assessing the prognosis of IBDs is of clinical interest.

The etiology of UC and CD is still unknown; however, microbial agents, immune dysfunction, genetic susceptibility, and environmental factors, such as diet, are implicated as pathogenic factors^3^. Luminal antigens induce abnormal immune responses, including intestinal inflammation, reactive oxygen species production, and circulation of inflammatory mediators, such as cytokines^3^. Inflammation localized in the intestine wall may radiate to the surrounding visceral adipose tissue, resulting in the proliferation and activation of adipocytes, especially in CD^4^. The activation of adipocytes releases subsets of fat-derived hormones, such as vaspin and adiponectin. These hormones influence gastrointestinal immune responses by modulating the expression of proinflammatory interleukins and adhesion factors, especially, TNF-α, which plays a crucial role in the pathogenesis of IBD^5^.

Lipids are increasingly recognized as key components of multiple signal transduction cascades, including those associated with the regulation of inflammation. The levels of free fatty acids, especially poly-unsaturated fatty acids (PUFAs), are dysregulated in the serum of patients with IBD. The levels of n-3 and n-6 PUFAs, precursors of eicosanoids that are critical in modulating inflammation, are positively correlated with disease severity and pro-inflammatory cytokine levels^6–9^.

Palmitoleic acid (PO) is a non-essential omega-7 monounsaturated free fatty acid. It is the third most common unsaturated free fatty acid in the human serum, after oleic acid (OA) and palmitic acid (PA). PO is an adipose tissue-derived lipid hormone (lipokine) that improves insulin sensitivity^10–14^. The role of PO in immunity remains largely unexplored; however, several studies have shown that PO mainly influences anti-inflammatory responses. Incubation with PO has been reported to reverse the expression of proinflammatory factors and secretion of cytokines during proinflammatory polarization in bone marrow-derived macrophages obtained from high-fat diet-fed mice^15^. PO reduces nuclear factor-kappa B phosphorylation and downregulates interleukin (IL)-6 and tumor necrosis factor (TNF)-α expression in hepatocytes and inflammatory cells^16^. Nevertheless, Wang et al. used cross-phenotype analysis of genome-wide association studies to show that high serum PA levels could be associated with an increased risk of developing CD. They also showed that PO tended to increase neutrophil recruitment in 2,4,6-trinitrobenezene sulfonic acid-induced colitis in zebrafish^17^. High serum PO levels have been observed in diseases involving metabolic dysregulation and inflammation, such as non-alcoholic steatohepatitis, hypertension, and heart failure^12,18^. Thus, PO could be a potential clinical marker for inflammatory diseases.

There is a growing interest in the role of fatty acids in metabolic and inflammatory diseases; however, the association between serum PO levels and IBD severity and prognosis remains unexplored. In this study, we aimed to elucidate the role of PO levels in the serum of patients with IBD and its potential for use as a clinical biomarker for IBD.

## Results

### Patient characteristics

The patient characteristics are summarized in Table 1. Among the study participants, 22 patients (12 men and 10 women) had UC, 35 (24 men and 11 women) had CD, 7 (3 men and 4 women) had colon cancer, and 22 (17 men and 5 women) were healthy controls. Patients in the UC group (median, 45 years; range 19–66 years) were significantly older than those in the CD (median, 32 years; range 17–55 years) and control (median, 27 years; range 22–54 years) groups. Patients in the colon cancer group (median, 67 years; range 52–87 years) were older than those in the UC and CD groups. The body mass index (BMI) of the patients in the CD group (median, 19.1; range 16.0–27.1) was significantly lower than that of the controls (median, 21.9; range 18.0–26.9).

**Table 1:** Clinical characteristics of the patients in this study.

	CD	UC	CC	NC
No.	37	25	7	22
**Sex**				
Male	26	14	3	17
Female	11	11	4	5
Age (range; in years)	32 (17–55)	45 (19–66)	67 (52–87)	27 (22–54)
BMI (range; in kg/m^2^)	19.1(16.0–27.1)	21.9 (13.7–36.9)	20.6 (19.6–23.2)	21.9 (18.0–26.9)
**CDAI**				
≥ 150	12	–	–	–
< 150	25	–	–	–
**CAI**				
≥ 6	–	11	–	–
< 6	–	14	–	–
**Current treatment**				
5-ASA	12	17	–	–
Steroid	5	12	–	–
Azathioprine	6	7	–	–
Infliximab	13	0	–	–
GCAP/LCAP	1	2	–	–
Tacrolimus	0	2	–	–
Elemental diet	15	0	–	–

*CD* Crohn’s disease, *UC* ulcerative colitis, *CC* colon cancer, *NC* normal control, *BMI* body mass index, *CDAI* Crohn’s disease activity index, *CAI* clinical activity index, *5-ASA* 5-aminosalicylic acid, *GCAP* granulocytapheresis, *LCAP* leukocytapheresis.

Disease status and treatment at the time of sample collection are shown in Table 2. Twelve patients with CD had a Crohn’s disease activity index (CDAI) score of ≥ 150, and 11 patients with UC had a clinical activity index (CAI) score of ≥ 6. Infliximab and 5-ASA were the most common treatments used by patients with CD, and steroid immunosuppressants and 5-ASA were the most common treatments used by patients with UC.

**Table 2:** Cox-hazard model showing the hazard risk of surgical procedures in patients with CD.

	HR	P	95% CI
PO µg/ml (by 10 µg/ml)	1.2782499	0.0244	1.0544814–1.663221
Previous surgery	0.9727294	0.9801	0.1109397–8.5289769
Use of anti-TNF-α agents	0.8883578	0.8852	0.1780416–4.4325586
Disease duration (months)	0.996789	0.6553	0.981503–1.010695
Age	0.977114	0.6976	0.860438–1.095513
BMI	0.834017	0.2558	0.567761–1.096203
Female sex	2.6849704	0.3168	0.3882601–18.567622
CRP	1.273887	0.0994	0.95395–1.7419

*CI* confidence interval, *PO* palmitoleate acid, *BMI* body mass index, *CRP* C-reactive protein.

### Serum PO levels in patients with IBD

**Figure 1:**
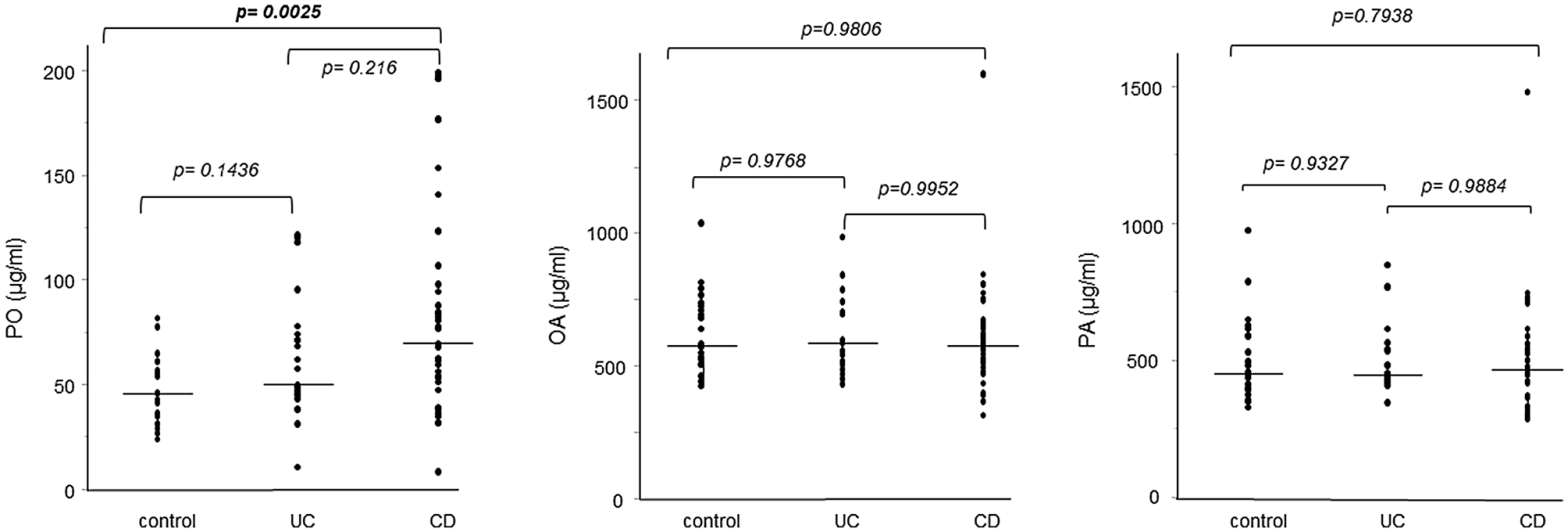
Elevation of serum palmitoleic acid (PO) levels in the serum of patients with Crohn’s disease (CD). Serum PO, oleic acid (OA), and palmitic acid (PA) levels were compared between patients with CD and healthy controls (control). The p-values in the graph show significant differences determined using the Steel–Dwass test.

Serum PO levels in the UC, CD, and control groups are shown in Fig. 1. Serum PO levels were significantly higher in patients in the CD group than those in the control group, whereas these levels were not significantly different between the UC and healthy control groups. In contrast, serum levels of OA and PA, the two major free fatty acids, did not differ among the control, UC, and CD groups.

**Figure 2:**
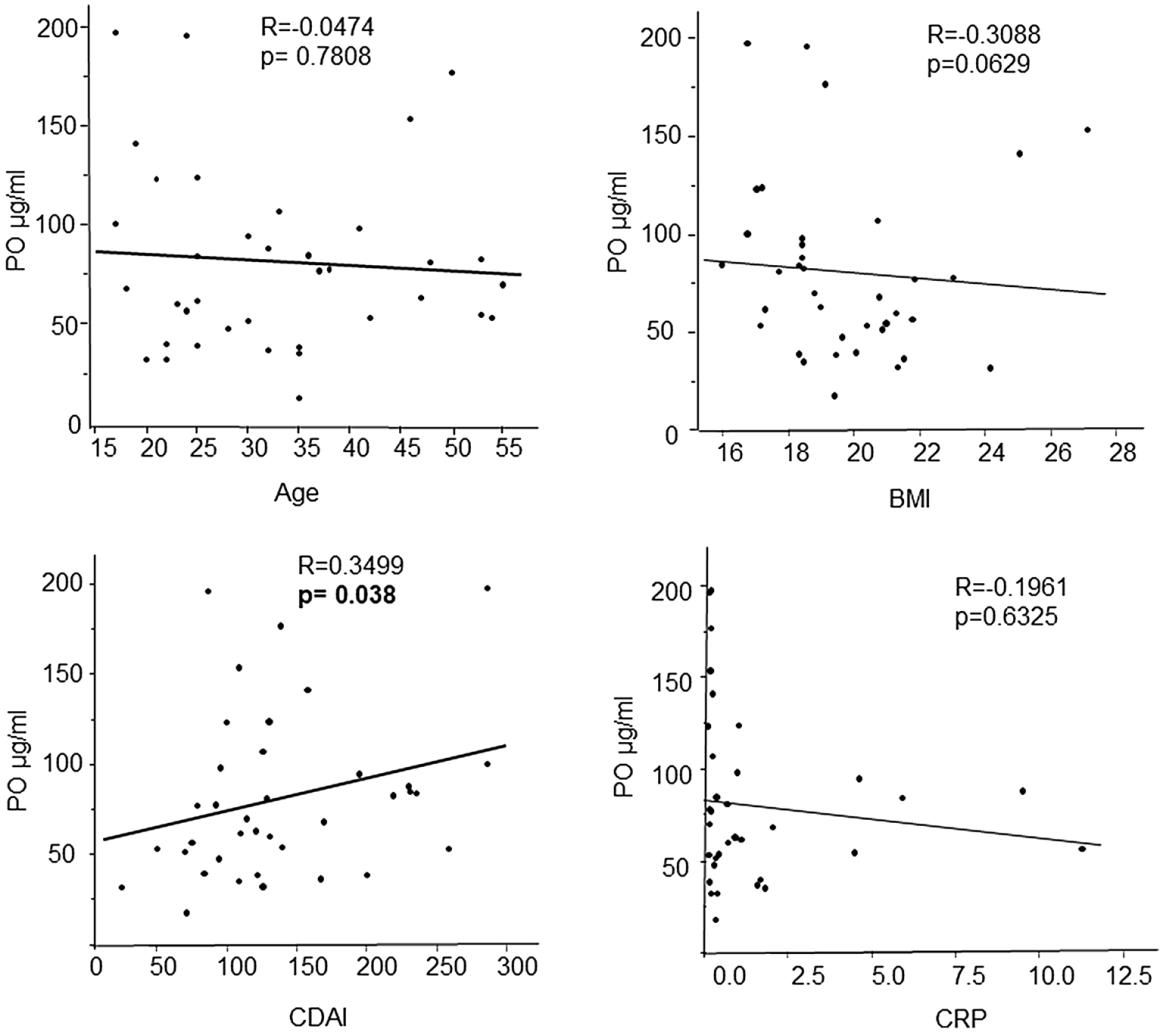
Serum palmitoleic acid levels correlate with Crohn’s disease activity index (CDAI) scores in patients with Crohn’s disease (CD). Correlation between palmitoleic acid (PO) levels and age, body mass index (BMI), C-reactive protein (CRP), or the Crohn Disease Activity index (CDAI) score in patients with CD. The p-values in the graph show significant differences determined using the Spearman’s test.

**Figure 3:**
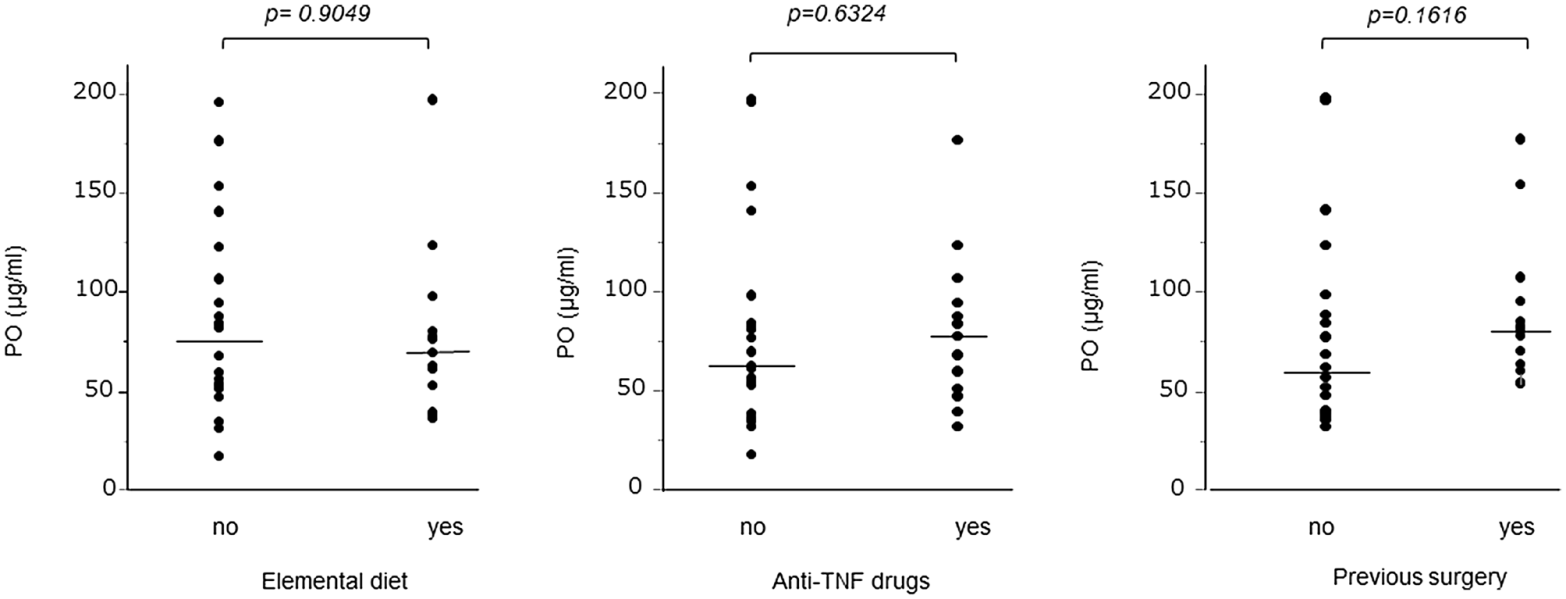
Serum palmitoleic acid (PO) levels are not affected by the history of elemental diet, surgery, or TNF-α treatment in patients with Crohn’s disease (CD). Comparison of serum PO levels among patients with CD based on the following factors: elemental diet, surgery history, and anti-TNF-α treatment. No significant difference between groups was observed based on the Wilcoxon test.

Based on these results, we assessed the factors that might affect PO level in patients with CD. Age, BMI, and C-reactive protein (CRP) levels did not have a significant correlation with PO levels, whereas the CDAI, which reflects disease severity, showed a moderate positive correlation with PO levels (Fig. 2). Ongoing treatment at the time of serum collection, such as the use of an elemental diet or anti-TNF-α agents and history of disease-associated surgery, did not significantly affect the levels of PO in the serum (Fig. 3).

### Association between PO and CD prognosis

Next, we assessed whether PO levels are related to IBD prognosis. This assessment involved patients who underwent surgical intervention after sample collection for multivariate Cox regression modeling (Table 2). Of the 35 patients with CD, 11 underwent surgical interventions, such as ileectomy, colectomy, abscess drainage, and fistula resection, during the follow-up. The follow-up period was 8–4080 days. PO was an independent risk factor for surgical intervention. Other factors, such as age, sex, anti-TNF-α agent use, CRP levels, and history of prior surgery, were not significantly associated with surgical intervention after serum collection (Table 2). The area under the receiver operating curve (AUC) for serum PO levels for individuals requiring surgical intervention was 0.73. When the appropriate baseline PO threshold was set at 68 µg/ml, based on the YOUDEN index, the sensitivity for predicting the need for surgical intervention was 72.1%, and the specificity was 75%.

### PO levels in the mesenteric adipose tissue

**Figure 4:**
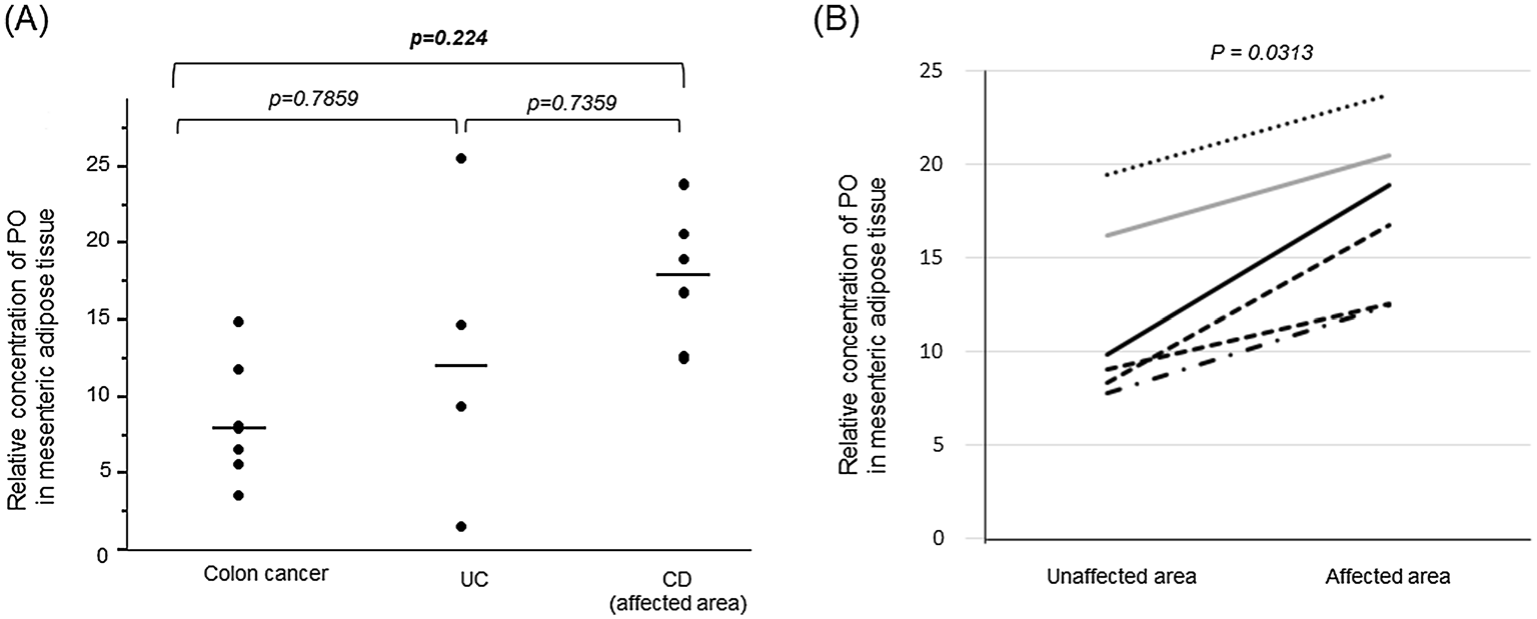
Palmitoleic acid (PO) levels increase in the mesenteric adipose tissue (MAT) of patients with Crohn’s disease (CD). (**A**) Comparison of PO levels in the MAT among patients with colon cancer, ulcerative colitis (UC), and CD. PO levels in the MAT in the disease-affected area. The p-values in the graph show significant differences determined using the Steel–Dwass test. (**B**) Comparison of PO levels in the MAT between unaffected and disease-affected areas in patients with CD. p = 0.0313, paired Wilcoxon test.

To investigate the potential source of serum PO, the levels of PO in the mesenteric adipose tissue (MAT) was studied in patients with IBD in a pilot study; patients with colon cancer (non-tumor area) were included in the control group. PO levels in the site of active disease in patients with CD were significantly higher than those in the MAT of patients with colon cancer (*p* < 0.0224, Fig. 4A). PO levels did not significantly differ in the MAT between patients with UC and those with colon cancer, showing a pattern similar to that in the serum. Within the CD group, PO levels were significantly higher in the active disease site than in the unaffected area (Fig. 4B).

## Discussion

In this study, we found serum PO levels to be significantly higher in patients with CD than in healthy controls; serum PO levels were positively correlated with disease activity in patients with CD but were not correlated with BMI or elemental diet, which are the possible factors besides disease activity that can affect PO levels. Elevated serum PO levels were associated with an increased risk of requiring surgical intervention.

PO has garnered attention in recent years because of its potential role as an adipokine^13,15,19^. Studies have indicated that circulating PO levels are associated with multiple metabolic risk factors and inflammatory diseases but in mixed directions, perhaps related to diversified lifestyle determinants or an endogenous source of fatty acids in humans^14,18,20^.

The lipid profile is altered in both the serum and adipose tissue in patients with IBDs^3,4,21^. A high ratio of PO to linoleic acid levels has been reported in children with CD^3^; its relevance to the clinical severity and course of CD in adults is poorly understood. To the best of our knowledge, the present study is among the first to analyze the detailed relationship between PO levels and IBD, with reference to disease activity and prognosis.

We observed elevated serum PO levels only in patients with CD, in comparison with its levels in the control subjects. The other two major free fatty acids, PA and OA, remained stable in the serum of patients with CD; therefore, PO could be a potential biomarker reflecting the disease status in CD. In addition, PO levels did not exhibit an association with BMI or elemental diet; therefore, PO levels do not merely reflect the nutritional status of the patients. Interestingly, circulating PO levels were associated with the CDAI as well as levels of CRP, which is a general inflammation marker. These results suggest that PO serves as an optimal biomarker for determining the clinical severity of CD. The CDAI score includes the severity of abdominal pain and incidence of diarrhea, the occurrence of which in patients must be recorded for 1 week. In addition, data comprising BMI and blood hematocrit levels are necessary, and physicians are required to confirm the absence or presence of abdominal mass. Therefore, serum PO level may be beneficial to estimate the inflammation state in patients with CD when the collection of necessary information is challenging. In addition, our study showed that PO levels serve as a significant indicator for the need of later surgical procedures, which might be helpful in identifying high-risk cases that are prone to drug therapy failure. Thus, PO levels could alert physicians regarding the risk of complicated CD, thereby aiding in deciding the extent of treatment and follow-up intervals.

The main sources of serum free fatty acids, including palmitoleate, include the adipose tissue, the liver, and dietary intake^19,22^. Accumulating evidence suggests adipose tissue in the mesentery might play a highly active role in the pathogenesis of CD^23^. For example, mesenteric adipocytes in patients with CD have been shown to secrete both proinflammatory mediators (e.g., TNF-α) and anti-inflammatory cytokines (e.g., interleukin-10)^23–25^. In CD, a high visceral to subcutaneous fat area has been reported as a biomarker for complications of CD including fistula and luminal stricture, which are often the indicators of surgery requirement. Our pilot study conducted using abdominal MAT samples from a small number of patients indicated a tendency of PO levels to increase in the active disease site of CD compared with that in patients with UC and controls. These findings suggest that PO may be released from the MAT. The possible explanation for the differential PO levels between patients with UC and CD is that inflammation caused by CD often affects the entire wall of the digestive system, which causes fat wrapping of the mesenchymal tissue as a defense mechanism in the active disease site^23,26^. A study in a larger population, including the other potential sources of PO, such as the liver and subcutaneous fat, could substantiate our hypothesis.

Limitations of this study include the relatively small number of individuals studied and the lack of functional studies of PO.

In general, PO supplementation is beneficial in suppressing inflammation, such as atherosclerosis in mice, where it prevents the activation of endoplasmic stress and inflammasomes^27^. PO blocks the inflammatory cascade triggered by endotoxins by downregulating the expression of Toll like receptor-4 (TLR-4) and nuclear factor-kappa B (NF-κB) and decreasing the production of pro-inflammatory cytokines in cultured primary macrophages^28^. PO treatment has been reported to be beneficial for patients with UC; however, there are no data on its use in patients with CD^29^. A previous study showed that the occurrence of SNPs near fatty acid desaturase 1 and glucokinase regulator was related to both increased serum PO levels and enhanced risk of CD in humans, thus indicating that high PO levels may be associated with an increased risk of CD. Our result is consistent with that of a previous study; it indicates that serum PO levels may also affect disease outcomes^17^.

In conclusion, our data indicate altered PO metabolism in CD and its role in identifying disease activity. PO has the potential to serve as a disease activity marker for local inflammation, as well as a prognostic factor, in CD.

## Materials and methods

### Study group

The protocol of this retrospective study was approved by the medical ethics committee of Nagasaki University Hospital Clinical Research Ethics Committee (approval no. 13040149). Patients who did not provide informed consent were excluded from the study. For subjects under 18 years of age, informed consent was obtained from their parents and/or legal guardian. This study was performed in accordance with the ethical guidelines of the Declaration of Helsinki. The serum study group consisted of 57 patients with IBD (35 patients with CD and 22 with UC) from April 2010 to March 2011. Twenty-two healthy volunteers were recruited as controls. The diagnosis of UC or CD was confirmed according to standardized criteria by prior clinical assessment, endoscopy, and histology. Omental fat specimens were obtained from 6 patients with CD and 4 with UC and were compared with those from 7 patients with colon cancer who underwent surgical resection between April 2013 and November 2014. The clinical records of the patients were then investigated from the time of sample collection until September 2020. Baseline data collected included those for sex, age, disease duration, surgical history, concomitant medications, CRP level, CAI, and CDAI. The dates of all surgical intervention procedures, including colectomy, enterectomy, and abscess drainage during the follow-up, were recorded for patients with IBD.

### Measurement of PO levels in the serum and MAT

Serum samples were collected from all patients with IBD and healthy volunteers in ethylenediaminetetraacetic acid tubes and preserved at − 80 °C until use. The MAT was collected during the colectomy. All surgeries were performed because of clinically necessary reasons. The MAT samples were stored at − 80 °C until use. Samples were homogenized in a Fast Prep System with Lysing Matrix D (MP Biomedicals, Solon, OH, USA). The homogenates were extracted using a mixture of methanol, chloroform, and purified water. The extracts were concentrated to complete dryness by blowing nitrogen. After transmethylation with HCl-methanol (3 h at 70 °C), the fatty acid composition was analyzed by gas chromatography (Agilent 7890B Gas Chromatograph, Agilent Technologies, Santa Clara, CA, USA) using a GC flame ionization detector system (Shimadzu, Tokyo, Japan) equipped with a capillary column (Omegawax 320; length, 30 m; internal diameter, 0.22 mm; film, 0.25 μM, Sigma-Aldrich, St. Louis, MO, USA) by TORAY Research Center (Shiga, Japan). Methyl stearate was used as the internal standard for free fatty acid analysis. The injector temperature was 250 °C, and the detector temperature was 270 °C. Initially, the column temperature was set to 50 °C for 1 min, and then ramped up to 270 °C at a rate of 8 °C/min. Helium was used as carrier gas at a flow rate of 30 cm/s. The concentration of fatty acids was assessed through a comparison with the standard Supelco 37 FAME component (37-component FAME mix, Sigma-Aldrich Japan, Tokyo, Japan).

### Statistical analyses

Statistical analyses were performed by a statistician (HK). Differences in quantitative values between two groups were assessed using the Wilcoxon rank-sum test. Differences in values among three or more groups were assessed using the Steel–Dwass test. A Cox proportional hazards model with person-days as the underlying metric was employed to estimate hazard ratios for surgical interventions for CD using the PHREG procedures in the SAS software package (version 9.4; SAS Institute, Inc., Cary, NC, USA). The AUC and cut-off value of PO levels for surgical interventions were calculated using the LOGISTIC procedure with the SAS software package. All statistical tests were two-sided, and results with p < 0.05 were considered statistically significant.

### Ethics declarations

This study was performed in accordance with the ethical guidelines of the Declaration of Helsinki.

### Consent to participate/consent to publish

Written informed consent was obtained from each patient.

## Data Availability

All data generated or analyzed in this study are included in this published article.
